# Potentiometric Chloride Ion Biosensor for Cystic Fibrosis Diagnosis and Management: Modeling and Design

**DOI:** 10.3390/s23052491

**Published:** 2023-02-23

**Authors:** Annabella la Grasta, Martino De Carlo, Attilio Di Nisio, Francesco Dell’Olio, Vittorio M. N. Passaro

**Affiliations:** Department of Electrical and Information Engineering, Polytechnic University of Bari, 70125 Bari, Italy

**Keywords:** biosensor, potentiometric sensing, cystic fibrosis

## Abstract

The ion-sensitive field-effect transistor is a well-established electronic device typically used for pH sensing. The usability of the device for detecting other biomarkers in easily accessible biologic fluids, with dynamic range and resolution compliant with high-impact medical applications, is still an open research topic. Here, we report on an ion-sensitive field-effect transistor that is able to detect the presence of chloride ions in sweat with a limit-of-detection of 0.004 mol/m^3^. The device is intended for supporting the diagnosis of cystic fibrosis, and it has been designed considering two adjacent domains, namely the semiconductor and the electrolyte containing the ions of interest, by using the finite element method, which models the experimental reality with great accuracy. According to the literature explaining the chemical reactions that take place between the gate oxide and the electrolytic solution, we have concluded that anions directly interact with the hydroxyl surface groups and replace protons previously adsorbed from the surface. The achieved results confirm that such a device can be used to replace the traditional sweat test in the diagnosis and management of cystic fibrosis. In fact, the reported technology is easy-to-use, cost-effective, and non-invasive, leading to earlier and more accurate diagnoses.

## 1. Introduction

The medical diagnostic field has undergone several stages of technological advancement, each building upon the previous developments. In the beginning, instruments were created to measure a wide range of analytes by collecting samples and transferring them to a laboratory. The next stage saw the advent of point-of-care testing (POCT), which brought the laboratory to the fingertips of healthcare professionals and even patients. Now, a new era is emerging where individuals can keep the lab with them using wearable biosensors for the real-time monitoring of biological markers [1,2]. Moreover, the integration of sensor technologies with mobile phones has given rise to a new field known as digital health, or mobile health (mHealth). mHealth has the potential to revolutionize healthcare by offering at-home diagnosis and patient management, as well as facilitating communication between patients and healthcare services. With the use of sensor technologies and big data analytics, the future of digital health holds the promise of a learning health system that will change the way diseases are managed and treatments are delivered.

Wearable technology presents a groundbreaking approach for addressing current medical issues. Thanks to their ability to constantly monitor both physiological and chemical biomarkers, as well as physical activities and behaviors, wearable devices have the potential to offer a comprehensive and real-time view of a person’s health [3,4,5].

The demand for wearable bioelectronics is increasing rapidly, and it has the potential to bring about a major transformation in the healthcare sector. Traditional medicine follows a reactive approach, where individuals are only diagnosed and treated after symptoms have already appeared. This has led to conventional medicine being referred to as “sick-care.” A shift towards a more proactive approach, where diseases can be detected and treated in their earliest stages, before symptoms even appear, is desirable. To make this happen, it is necessary to obtain a deeper understanding of the body’s function at the chemical and molecular level. This can be achieved by using wearable biosensors, through which it is possible to provide not only continuous and non-invasive monitoring of biological markers, but also personalized medicine, modified according to each person’s characteristics and lifestyle [6,7]. Another important advantage of such a technology is that it is not expensive or time-consuming, and it avoids the possibility of making even small mistakes, which can compromise the accuracy of a test.

Biosensors can be used to measure a wide range of bodily fluids, including blood, urine, saliva, and sweat. Each of these body fluids contains unique chemical and biological markers that can provide important information about an individual’s health.

One way to classify biosensors is based on their transduction mechanisms, namely, mechanical, optical, and electrochemical biosensors. Specifically, our focus is on electrochemical sensors, which strictly rely on the chemical interactions between a biorecognition layer and the target biomolecules to be measured, and which can be differentiated into three different categories, including amperometric biosensors, impedimetric biosensors, and potentiometric biosensors [8].

Potentiometric biosensors, which can detect the potential difference determined between a reference electrode and an indicator electrode with a very high impedance voltmeter, in almost zero current flow conditions, are currently the subject of growing scientific interest due to their very high sensitivity and specificity obtained when an extremely stable reference electrode is used [9,10,11,12,13,14].

In recent years, numerous potentiometric biosensors, even wearable sensors, have been developed for the monitoring of different biochemical substances, exploiting the activity of bioreceptors including enzymes, antibodies, and nucleic acids. Seema et al. [15] prepared nanoparticles of urease from jack beans and developed a potentiometric biosensor to detect urea in human urine samples. Canovas et al. [16] developed a potentiometric biosensor for the direct detection of glucose in whole, undiluted blood without any sample pre-treatment. Mishra et al. [17] fabricated a wearable potentiometric tattoo biosensor for real-time on-body monitoring of G-type nerve agents simulants. Bandodkar et al. [18] described the fabrication of an epidermal temporary-transfer tattoo-based potentiometric sensor, coupled with a miniaturized wearable wireless transceiver, for real-time monitoring of sodium in human perspiration. Papp et al. [19] fabricated a potentiometric biosensor for detecting tumor, hepatitis B, digoxin, and troponin I.

Among potentiometric biosensors, particular relevance is assumed by those based on field effect transistors, mainly studied for sensing applications, as for example the ion-sensitive field-effect transistor (ISFET). This device was invented in 1970 [20] and was first used for the sensing of pH and later for the measurement of the concentration of a plurality of biomarkers of biomedical interest, including potassium iodide (KI), ethanol, serum glucose, cholesterol, and hydrazine [21]. ISFET has many advantages in the field of monitoring electrochemical reactions; in fact, it can be produced on a large scale and integrated into other detection systems; moreover, it is beneficial in terms of size, due to the presence of only one reference electrode. As a solid-state sensor, without fragile materials involved, it is acids-alkali resistant, water repellent, and shock resistant. Currently, after the possibilities of clinical detection have been verified, the research emphasis has shifted to the technique for improving the performance of the ISFET biosensors. The related studies mainly include the material, structure, and functionality of the gate.

Over the past few years, ISFETs have rapidly progressed and become one of the most widely used biochemical sensors, with hundreds of related papers being published annually. The application is not limited to single detection. By using ISFET arrays, multiplexed detection with a single chip can also be achieved because ISFETs are easily integrated. By integrating multiple ISFETs onto one sensing chip, the on-off state of each one can be controlled electrically. Only the “on” ISFET will operate and transmit biochemical signals from the corresponding analytes as electrical signals. By operating the ISFETs with different functionalities one by one, it is possible to detect different analytes simultaneously with a single chip. Additionally, by fabricating ISFETs in a small size, with high performance, portable and wearable devices can be developed [22].

The ISFET architecture is similar to that of a MOSFET, with the difference being that the gate metal is removed in such a way as to establish a direct contact between the insulating surface and the electrolytic solution to be tested; in particular, it takes advantage of the fact that the current between the source and the drain is modulated by the potential of the gate, which in turn depends on the concentration of the analyte that binds to the sensitive material.

The clinical case of interest for which the implementation of an ISFET has been carried out is cystic fibrosis, a disease generally diagnosed and monitored through a conventional exam-defined sweat test. The distribution of sweat glands in the human body is rich (>100 glands/cm^2^), and the sweat contains abundant biochemical compounds; thus, human sweat has become a promising avenue for non-invasive biosensing. This is because the widespread presence of eccrine glands on human skin makes this biofluid easily accessible, without having to resort to painful or invasive devices, such as needles. Sweat is a biological fluid that is secreted by the skin through the transepidermal water loss (TEWL), which is regulated by the diffusion of water vapor in the stratum corneum due to a concentration gradient that occurs between the inner and the outer surface of the epidermis. Produced by the sweat glands, perspiration is therefore the main vehicle for the transport of salts through the body. Under normal conditions, the sweat that evaporates and reaches the surface of the skin is slightly salty. Subjects with cystic fibrosis have a mutation in the gene encoding the chloride conductive transmembrane channel, called the cystic fibrosis transmembrane conductance regulator (CFTR), which regulates the transport of water and salts inside and outside the cells; as a result, the epithelial tissue fails to absorb chloride, at the same time causing insufficient sodium adsorption from the ducts. When this is achieved, NaCl concentrations above 70 mol/m^3^ are found on the skin surface [23], which is why sweat will be heavily salted.

The concentration of sodium and chloride ions in sweat can vary depending on several factors, including hydration status, diet, and overall health. However, there are some general norms for the concentration of these ions in human sweat. Typically, the concentration of sodium ions in sweat ranges from 50 to 200 mM/L. The concentration of chloride ions in sweat is typically similar to that of sodium, ranging from 40 to 140 mM/L. These values can vary depending on the individual and the specific conditions under which the sweat was produced.

When sweat analysis is carried out through conventional testing, diagnostic errors may occur that could compromise the outcome of the diagnosis of cystic fibrosis; the use of an ISFET for the monitoring of salts in the sweat of patients subject to such pathology could be useful to optimize the clinical trial, minimizing the number of false positives, ensuring continuous and real-time monitoring, and checking in advance whether or not the therapy administered is effective.

This paper reports on the modeling and design of an ISFET-based potentiometric biosensor intended for ${\mathrm{Cl}}^{-}$ sensing in sweat. The paper is organized as follows: it starts with the description of the configuration and operating principle of the designed ISFET, explaining the analogy with respect to MOSFETs and the theories which explain the chemical reactions that take place between the gate oxide and the electrolytic solution; then, the modeling of the device through the finite element method is reported by describing the equations referred to in the two adjacent but distinct domains: the electrolyte domain and the semiconductor domain; finally, the obtained numerical results are presented.

## 2. ISFET-Based Sensor: Configuration and Operating Principle

The ISFET is a solid-state potentiometric sensor that uses the principle of an FET to convert a concentration of charged ions into an electrical signal. Its structure is very similar to that of a MOSFET, but it is modified with respect to the latter by replacing the gate with an electrolyte solution containing the target ions to be measured, along with a reference electrode. In this way, the electric potential at the gate oxide–solution interface depends on the concentration of ions in the electrolyte.

Due to the presence of the electrolyte solution between the reference electrode and the insulator, the expression of the threshold voltage in an ISFET is different from that of a MOSFET, and it may be written as follows [24]:

(1) $$V_{TH}=E_{ref}-\psi_{0}+\chi^{sol}-\phi_{Si}-\frac{Q_{ox}+Q_{ss}+Q_{B}}{C_{ox}}+2\phi_{f}$$

where $E_{ref}$ is the reference electrode potential relative to vacuum, $\psi_{0}$ is the electrostatic potential, $\chi^{sol}$ is the surface dipole potential of the solution and it is a constant, $\phi_{Si}$ is the silicon work function, and $C_{ox}$ is the gate insulator capacitance per unit area. Other parameters that affect the value of the threshold voltage are the charges located in the oxide ($Q_{ox}$), the surface states and interface states ($Q_{ss}$), and the depletion charge ($Q_{B}$).

$\phi_{f}$ is the potential difference between the Fermi levels of doped and intrinsic silicon, given as:

(2) $$\phi_{f}=\frac{kT}{q}ln\left(\frac{N_{A}}{n_{i}}\right)$$

where k is the Boltzman constant, T is the absolute temperature, q is the elementary charge, $N_{A}$ is the acceptor concentration, and $n_{i}$ is the intrinsic carrier concentration of silicon. Except for the electrostatic potential, $\psi_{0}$, all the parameters in the threshold voltage expression remain the same, regardless of the ions’ concentration in the electrolyte solution [25]. As a result, the variation in the ions’ concentration is the only thing that can be credited with the changes that occur in the $V_{TH}$ expression.

**Figure 1:**
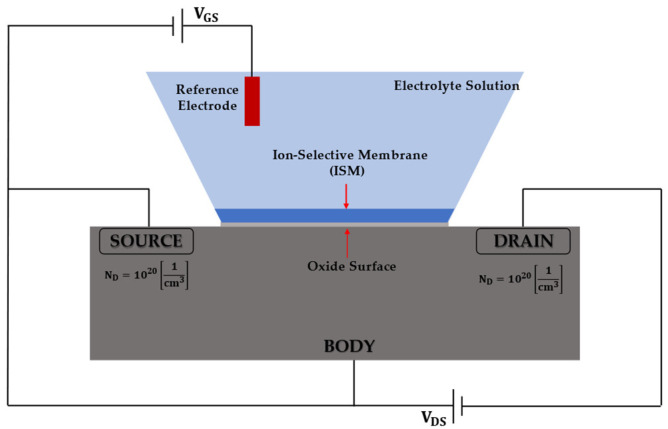
Schematic diagram of the designed ISFET.

In Figure 1, the sensor cross-section is shown, and for its modeling, we have considered that the body of the semiconductor is 0.7 μm in height, and its width is 3 μm. The source and drain are 0.5 μm in length towards the gate. The gate region is 1.3 μm wide and is large enough to act as a reacting surface to the electrolyte solution.

The selectivity of the ISFET is primarily determined by the ion-sensitive membrane, which makes the sensor responsive only to the specific target ions.

Since the ISFET is not water repellent, its encapsulation is necessary. Encapsulation techniques for ISFETs are critical to ensuring stable and consistent electrical properties. Some encapsulation techniques that can minimize variations in electrical properties are described in the literature. For example, the dam-and-fill technique is widely used in ISFETs encapsulation, and it guarantees a small degradation of the sensor performance [26,27]. Thus, the performance prediction achieved by our accurate model is realistic, despite the fact that we have modeled the sensor without considering encapsulation.

We assume that the sensor is always polarized in the saturation operating region. In this way, any $V_{TH}$ change due to variations in the ion concentration in the electrolyte solution induces a change in the drain-source current, $I_{DS}$, which is given by [24]:

(3) $$I_{DS}=K{\left(V_{GS}-V_{TH}\right)}^{2}$$

(4) $$K=\frac{1}{2}(\mu_{n}C_{OX}\left(\frac{W}{L}\right))$$

where $\mu_{n}$ is the electron mobility, $C_{OX}$ is the oxide capacitance per unit area, and W and L are the width and the length of the channel, respectively.

**Figure 2:**
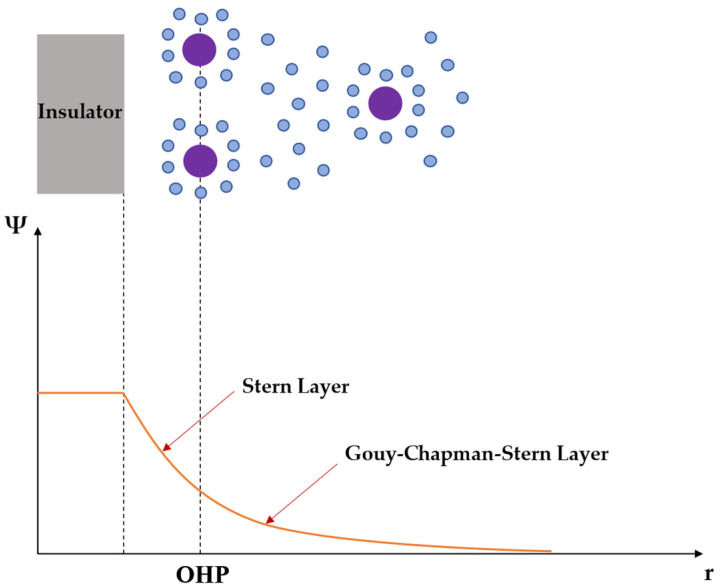
Illustration of the Stern layer.

The chemical reactions that take place between the gate oxide and the electrolytic solution can be described according to two major theories [25,28,29,30]:

(a) The site-binding theory, which analyzes the mechanism of oxide surface charge creation, considering the equilibrium between the amphoteric surface sites and ions in the solution;
(b) The Gouy–Chapman–Stern theory, which models ion distribution in the electrolyte solution through different layers, such as the Helmholtz layer and the diffuse double layer, where the first one contains the ions which are strongly adsorbed by the electrode surface, and the second one contains the ions which are distributed with a concentration gradient driven by the thermal motion. In particular, Stern observed that ions cannot approach the electrode surface closer than their ionic radius, and the distance of the closest approach is called the outer Helmholtz plane (OHP). The occurrence of this phenomena is illustrated in Figure 2. It should be noted that the solvation shell of water molecules also contributes to this distance of closest approach. To break the ion free from its watery prison, a greater quantity of energy will be necessary. Because of this, an area adjacent to the electrode surface will be devoid of any ionic charges, and this will give rise to a constant capacitance, known as Stern capacitance, which typically has a value of 20 μF/cm^2^ [31].

## 3. ISFET Modeling

The ISFET-based sensor is simulated and designed by using the finite element method (FEM), considering two adjacent domains:

- the electrolyte domain, obtained by approximating sweat as an aqueous solution at acid pH containing the ions of interest;
- the semiconductor domain, set up as a MOSFET.

Sweat pH is slightly acidic, usually between 4 and 6.5, so in our simulations, a pH of 6 has been considered.

### 3.1. Semiconductor Domain

In the semiconductor domain, Poisson’s equation for electric potential (5) and the drift-diffusion equations for electrons and holes in the semiconductor material (6) are solved [32]:

(5) $$\nabla \cdot \left(-\in_{0}\in_{r}\nabla V\right)=\rho$$

(6) $$\begin{array}{c} J_{n}=qn\mu_{n}\nabla E_{c}+qD_{n}\nabla n-qnD_{n}\nabla ln\left(N_{c}\right)+qnD_{n,th}\nabla ln\left(T\right)\\ J_{p}=qp\mu_{p}\nabla E_{V}-qD_{p}\nabla p+qpD_{p}\nabla ln\left(N_{v}\right)-qpD_{p,th}\nabla ln\left(T\right) \end{array}$$

where $\in_{0}$ and $\in_{r}$ are the absolute and relative permittivity, ρ is the charge density, q is the elementary charge, n is the number of electrons, $\mu_{n}$ is the electron mobility, $E_{c}$ is the minimum conduction electron energy, $D_{n}$ is the diffusion coefficient for electrons, $N_{c}$ is the effective density of states (conduction band), and T is the temperature. All parameters are defined in the same manner for the drift-diffusion equation of the holes.

### 3.2. Electrolyte Domain

The formation of the diffuse double layer is treated by combining:

- the electrostatic physics, hence, the Poisson’s equation for the charge density and the electric field;
- the physics of the transport of chemical species within a solution, thus, the Nernst–Planck’s equation for the mass transport of ions:

(7) $$J_{i}=-D_{i}\nabla c_{i}-u_{m,i}z_{i}Fc_{i}\nabla \varphi_{l}\;$$

where $D_{i}$, $c_{i}$, and $z_{i}$ refer to the diffusion coefficient, the concentration, and the charge number of the i-th ionic species, F is the Faraday constant, and $\varphi_{l}$ is the electric potential of the electrolytic phase. Equations (5) and (7), characterizing the two physics above mentioned, are coupled through the common variable $\varphi_{l}$.

The mobility is calculated as a function of the diffusion coefficient of the ionic species and the temperature, using the Nernst–Einstein’s equation [33]:

(8) $$u_{m,i}=\frac{D_{i}}{RT}$$

where R is the molar gas constant.

Assuming that there are no homogeneous reactions of ions in the solution, for mass preservation, the following condition must be satisfied:

(9) $$\nabla \cdot J_{i}=0$$

In our case, we considered the transport of four ionic species, namely $H^{+}$, ${\mathrm{OH}}^{-}$, ${\mathrm{Na}}^{2+}$, and ${\mathrm{Cl}}^{-}$. At this point, since in cystic fibrosis, the major effects are caused by the adsorption of negative ions [34], i.e., chloride, ${\mathrm{Cl}}^{-}$, a modified version of the site-binding model must be considered (see Equation (10)), which consequently produces a variation in the Boltzmann equation (11) [34]:

(10) $$HfOH_{2}^{+}+Cl^{-}\Leftrightarrow HfOHCl^{-}$$

(11) $$\left[Cl_{S}^{-}\right]=\left[Cl_{B}^{-}\right]\exp\left(\frac{q\psi_{0}}{kT}\right)$$

where $\left[Cl_{S}^{-}\right]$ and $\left[Cl_{B}^{-}\right]$ represent the concentration of chloride ions on the insulating surface and in the electrolyte, respectively, q is the elementary charge, $\psi_{0}=\varphi_{M}-\varphi_{l_{bulk}}$ is the surface potential, and k is the Boltzmann constant. The effect of the interface between the reference electrode and the sample solution is taken into account with the silver electrode work function (WAg = 4.6 V).

### 3.3. Thin Oxide: Semiconductor Domain and Electrolyte Domain Coupling

**Figure 3:**
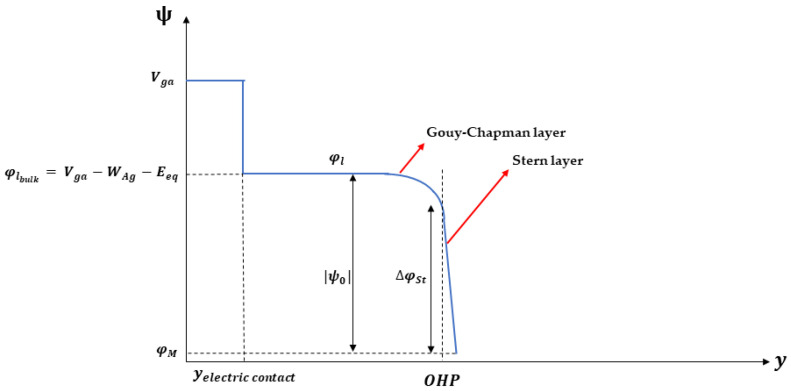
Potential diagram of ISFET along the vertical axis in the electrolyte domain.

At the level of the semiconductor domain, the oxide is modeled through the “thin insulator gate” boundary condition, which forces the potential to be $\varphi_{M}$-equal. The Bode module binds the latter to the electrolyte potential at the upper edge of the thin oxide, $\varphi_{l}$, through the equation:

(12) $$C_{st}=\frac{\sigma_{0}}{\varphi_{l\left(y_{ox-}\right)}-\varphi_{M}}$$

which therefore imposes the difference in potential *(*$\Delta \varphi_{St}=\varphi_{l\left(OHP\right)}-\varphi_{M}$) on the $C_{st}$ capacity, representing the Stern capacity, with the charge density on the electrodes. It is possible to obtain a clearer vision of the potentials mentioned in this paragraph by evaluating their trend along the vertical axis of the device, as shown in Figure 3; moreover, a detailed description of the parameters of the model is given in Table 1.

Equations (10) and (11) show the role of ${\mathrm{Cl}}^{-}$ ions at the oxide surface in determining the surface charge density, $\sigma_{0}$, which can be expressed by the following [29,35]:

(13) $$\sigma_{0}=qN_{S}\frac{{\left[H_{S}^{+}\right]}^{2}\left[H_{B}^{+}\right]-K_{a}K_{b}\left[H_{B}^{+}\right]-K_{c}\left[Cl_{S}^{-}\right]{\left[H_{S}^{+}\right]}^{2}}{{\left[H_{S}^{+}\right]}^{2}\left[H_{B}^{+}\right]+K_{a}K_{b}\left[H_{B}^{+}\right]+K_{c}\left[Cl_{S}^{-}\right]{\left[H_{S}^{+}\right]}^{2}+K_{b}\left[H_{S}^{+}\right]\left[H_{B}^{+}\right]}$$

where q is the elementary charge, $N_{S}$ represents the density of surface sites per unit area, $\left[H_{S}^{+}\right]$ and $\left[H_{B}^{+}\right]$ are the concentrations of hydrogen ions on the insulating surface and in the electrolyte, respectively, $K_{a}$ and $K_{b}$ are the dissociation constants for deprotonation and protonation reactions, while $K_{c}$ is the reaction constant for chloride, and it is a dimensionless quantity.

At the level of the electrolyte domain, the oxide is modeled through the definition of the “electric displacement field from semiconductor” boundary condition, which specifies the continuity of the normal component of the electric induction field at the boundary where the oxide is ideally located.

### 3.4. Ion-Selective Membrane (ISM)

When ISFETs are modified to be chemically sensitive to ions other than $H^{+}$, an ion-selective membrane, also known as an ISM, may be modeled and placed in various locations, such as above the oxide layer [36,37]. ISMs can either be in the solid (glassy or crystalline) or liquid phase. In the first case, ionic sites are present as part of the crystal structure and are covalently bound to the solid network, whereas in the liquid phases, they are usually added in the form of lipophilic salts [38]. However, solid state membrane selectivity cannot be easily tuned and is not flexible. The most prevalent types of ISMs are liquid membranes, typically made of polymeric materials that incorporate a microporous support matrix. This support should be chemically inactive and avoid the dispersion of all membrane components; the most common polymer used in ISMs is poly(vinylchloride) (PVC).

In our study, we have considered a PVC-ISM, with $l_{ISM}$ = 1.67 [μm] and $d_{ISM}\;$= 0.5 [μm], so as to better filter the flux of ${\mathrm{Cl}}^{-}$ ions from the electrolyte. This is achieved by using a selectivity coefficient, $K_{i}$, which is defined as the ratio between the concentration on the upside and on the downside of the boundary considered ($c_{i,u}$ and $c_{i,d}$, respectively):

(14) $$K_{i}=\frac{c_{i,u}}{c_{i,d}}$$

The selectivity coefficient allows for specifying at which ion the membrane must be more selective, so it acts as a multiplicative term, defined in a range between 0 and 1, which decreases the concentration of the *i*-th species from the sample solution into the ISM [39].

## 4. Numerical Results

**Figure 4:**
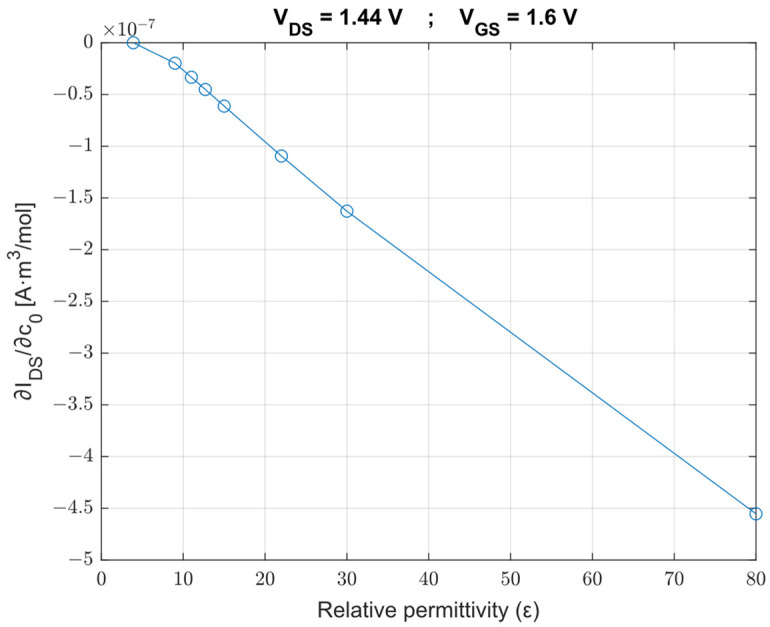
Drain-source current variation for two concentration values as a function of the *high*-k oxide used as an insulating layer.

As shown in Figure 4, we have analyzed the performance of the ISFET when *high*-k oxides (silicon dioxide, aluminum oxide, hafnium (IV) silicate, zirconium (IV) silicate, yttrium (III) oxide, tantalum (V) oxide, hafnium (IV) oxide, lanthanum (III) oxide, titanium dioxide) are used as the insulating layer, so we have examined the correlation between the derivative of the drain-source current with respect to the chloride ion concentration $c_{0}$ (sensitivity, *S*) and the relative permittivity. We can observe that as the permittivity increases, the derivative of $I_{DS}$ with respect to $c_{0}$ reduces.

**Figure 5:**
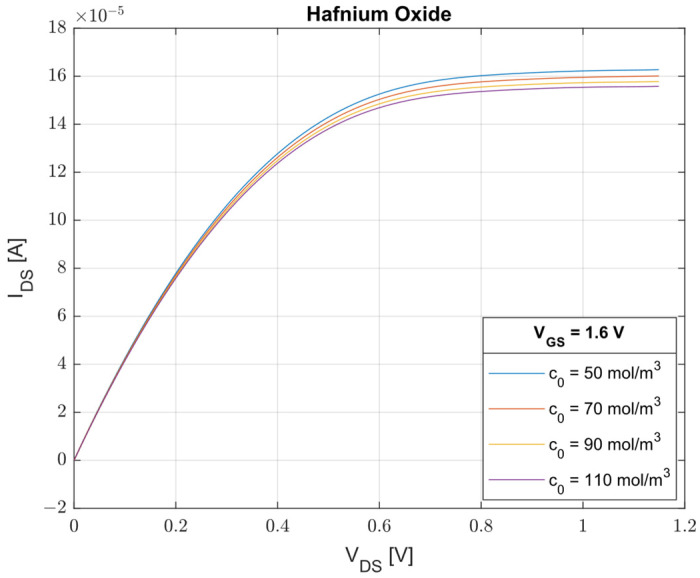
Optimal performances of ISFET: drain-source current vs. drain-source voltage for four different $\left[{\mathrm{Cl}}^{-}\right]$ values using HfO_2_ as the insulating layer.

The oxide that has shown better performances in terms of sensitivity ($S=1.2\times 10^{-7}\;\frac{A\cdot m^{3}}{\mathrm{mol}}$) is the Hafnium oxide ($\varepsilon_{ins}$ = 25, $d_{ins}$= 30 nm, $l_{ins}$ = 1.6 μm). In Figure 5, assuming $V_{GS}$ = 1.6 V, we have observed the evolution of the $I_{DS}-V_{DS}$ characteristics for four values of ${\mathrm{Cl}}^{-}\;$ concentration ($c_{0}$ = 50 mol/m^3^, $c_{0}$ = 70 mol/m^3^, $c_{0}$ = 90 mol/m^3^, $c_{0}$ = 110 mol/m^3^). From this result, we can state that the designed device proves to be successful for the management of cystic fibrosis, exhibiting a difference in saturation current, $\Delta I_{DS}$, of 7.8 μA when $c_{0}$ passes from 50 mol/m^3^ to 110 mol/m^3^. Moreover, assuming a minimum-detectable change in current, $\min\left(\Delta I_{DS}\right)$, of 0.5 nA [40], it is possible to obtain a corresponding resolution in concentration, $\left(\Delta c_{0}\right)$, of 0.004 mol/m^3^.

**Figure 6:**
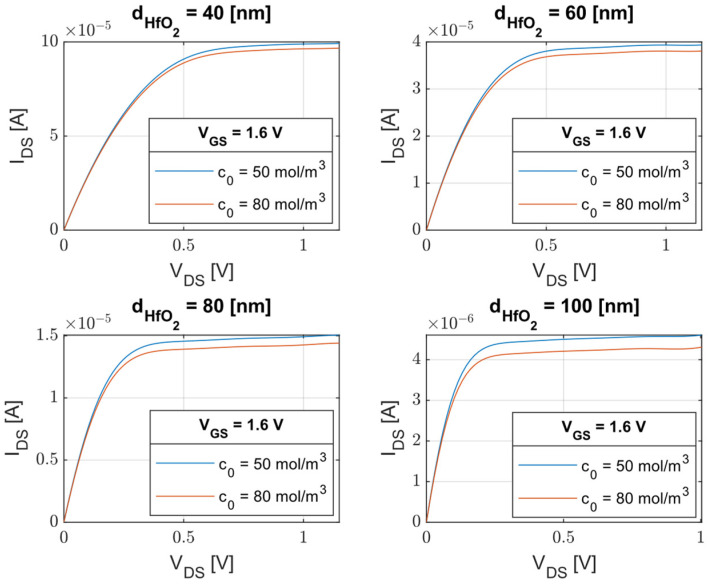
Drain-source current vs. drain-source voltage for two different $\left[{\mathrm{Cl}}^{-}\right]$ values using various values of HfO_2_’s thickness.

Another aspect that was thought to affect the performance of the device is the variation of its geometry, such as the width of the oxide. In Figure 6, always considering HfO_2_ as the insulator, it is possible to see how the dependence of the drain-source current, $I_{DS}$, on the drain-source voltage, $V_{DS}$, varies depending on four values of $d_{HfO_{2}}\;$ and, in particular, the sensitivity of the device reduces as the thickness increases. This agrees with the classical behavior of MOSFETs.

**Figure 7:**
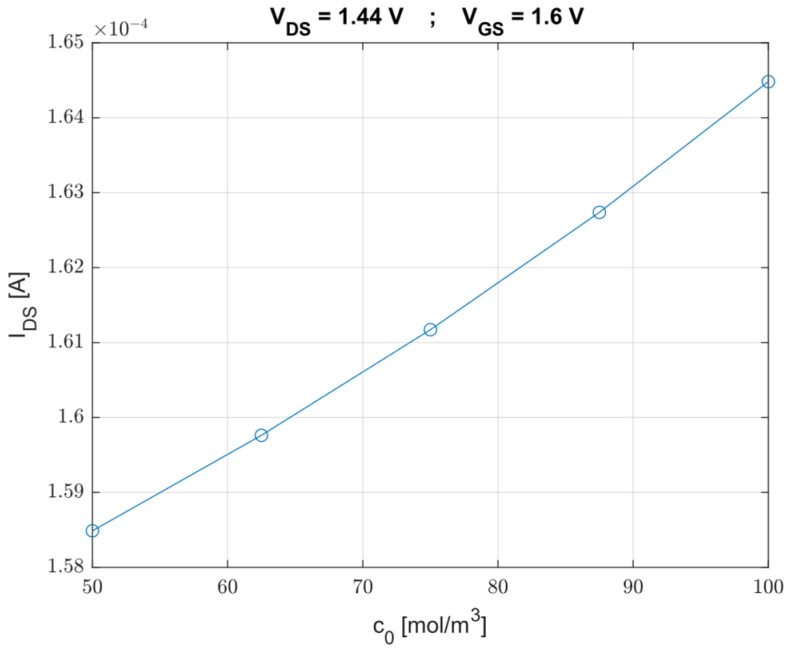
$I_{DS}/c_{0}$ curve with a fixed value of $V_{DS}$ for ISFET-based ${\mathrm{Cl}}^{-}$.

Then, we have calculated the drain-source current, $I_{DS}$, in function of five values of ${\mathrm{Cl}}^{-}\;$ concentration, with a fixed value of the drain-source voltage, $V_{DS}$ = 1.44 V, and of the gate-source voltage, $V_{GS}$ = 1.6 V. As shown in Figure 7, there is a direct correlation between these two parameters; in fact, the higher is the concentration we want to detect, the higher is the saturation current.

## 5. Conclusions

A potentiometric biosensor for chloride ion sensing in sweat has been modeled and designed, showing great promise for the monitoring of cystic fibrosis. The FEM-based modeling approach couples the physics of semiconductors with electrochemistry. The achieved results are promising towards the development of an ISFET-based ${\mathrm{Cl}}^{-}\;$ sensor for use in the context of point-of-care diagnosis, paving the way for the development of a compact and portable technology, replacing traditional diagnostic techniques. With further development and optimization, and its easy-to-use, cost-effective, and non-invasive nature, the ${\mathrm{Cl}}^{-}$-based ISFET has the potential to revolutionize the way the pathology is diagnosed and monitored.

## Figures and Tables

**Table 1:** Description of the potentials involved in the study.

Potentials	Description
$V_{ga}$	Gate voltage applied
$W_{Ag}$	Work function of Ag electrode
$E_{eq}$	Equilibrium potential of reference electrode
$\varphi_{l}$	Electrolyte potential
$\varphi_{l}{}_{bulk}$	Bulk electrolyte potential
$\varphi_{M}$	Potential imposed at the upper edge of the oxide
$\psi_{0}$	Difference in potential between $\varphi_{M}$ and ${\varphi l}_{bulk}$
$\Delta \varphi_{St}$	Difference in potential applied on $C_{st}$ capacity

## Data Availability

The data will be provided by the corresponding author under reasonable requests.
